# Monitoring the trends of water-erosion desertification on the Yunnan-Guizhou Plateau, China from 1989 to 2016 using time-series Landsat images

**DOI:** 10.1371/journal.pone.0227498

**Published:** 2020-02-05

**Authors:** Liang Hong, Yajun Huang, Shuangyun Peng

**Affiliations:** 1 College of Tourism and Geographic Science, Yunnan Normal University, Kunming, Yunnan, China; 2 GIS Technology Research Center of Resource and Environment in Western China of Ministry of Education, Yunnan Normal University, Kunming, Yunnan, China; 3 Center of Geospatial Information Engineering and Technology of Yunnan Province, Yunnan Normal University, Kunming, Yunnan, China; 4 Key Laboratory of Resources and Environmental Remote sensing for Universities in Yunnan, Yunnan Normal University, Kunming, Yunnan, China; University of Waikato, NEW ZEALAND

## Abstract

The Yunnan–Guizhou Plateau (YGP) is a typical ecologically fragile region in southwest China. Water-erosion desertification (WED) is one of the most significant environmental and socio-economic issues on the YGP and has seriously restricted the socio-economic development of this region. However, the research on monitoring of the desertification trends in this region has been limited to long time-series Landsat imagery. The objectives of this research were to monitor the WED trends on the YGP using time-series Landsat imagery data from 1989 to 2016. In this paper, we present a multi-indicator rule-based method, which was used to map the WED on the YGP during this period. The results show that the addition of multiple indicators improved the WED classification accuracy to 90.61%. Overall, the following results were obtained by using the proposed method. (1) The slight desertification area on the YGP increased from 89,617.09 km^2^ in 1989 to 100,976.47 km^2^ in 2016 with an annual growth ratio (AGR) of 0.48%, the moderate desertification area increased from 80,276.65 km^2^ in 1989 to 90,768.39 km^2^ in 2016 with an AGR of 0.50%, and the severe desertification area increased from 8149.3 km^2^ in 1989 to 13,220.16 km^2^ in 2016 with an AGR of 2.39%. (2) The WED expansion on the YGP can be divided into three stages. Firstly, the total WED area increased slowly from 17.80×10^4^ km^2^ in 1989 to 17.98×10^4^ km^2^ in 2010 with an AGR of 0.05%. Then, the WED rapidly expanded from 17.98×10^4^ km^2^ in 2010 to 20.28×10^4^ km^2^ in 2013 with an AGR of 4.26%. Finally, the WED increased slightly from 20.28×10^4^ km^2^ in 2013 to 20.50×10^4^ km^2^ in 2016 with an AGR of 0.36%. The total areas of the different degrees of WED decreased in 1992, 1998, 2001, and 2004. (3) The driving factors of WED were analyzed based on the Geographically Weighted Regression (GWR) model. We found that precipitation, vegetation area, and gross domestic product have key roles in the processes of desertification reversion and development. However, the regression coefficients between WED and these factors exhibited considerable spatial variations. The regression coefficients of the key driving factors showed different spatial distributions based on the GWR model in the YGP. The research results can provide scientific reference information for the prevention and control of WED in the YGP.

## Introduction

Water-erosion desertification (WED) is a special type of land desertification due to soil erosion [1–5], which is mainly distributed in the Loess Plateau and Yunnan–Guizhou Plateau (YGP). The WED in the karst regions is also called rocky desertification. The rocky desertification in karst regions means to the loss of surface soil due to soil erosion by rain or stripped by water. The main features are: serious soil erosion; extensive exposure of basement rocks; drastic decrease of soil productivity. According to the Fifth National Monitoring Survey results obtained by the Natural Forestry and Grassland Administrator, the area of WED on the YGP is about 251,100 km^2^, amounting to 9.58% of the total area [6–8]. However, the YGP is one of the largest karst-dominated regions in the world, which are typically ecologically fragile regions [9]. The WED has seriously harmed the ecological environment, natural resources, and socio-economic development on the YGP[10], so WED prevention and control are critically important and urgent in this area. Monitoring WED trends is an important and effective means of combating desertification on the YGP. Traditional WED monitoring methods rely on ground surveys, which are labor intensive, time consuming, and expensive and limit time series comparisons and regional-scale research[11]. With the development of remote-sensing technology, it has become possible to monitor desertification trends for long time series and large areas.

Over the past decades, because of the abundance of remote sensing images (e.g., Landsat TM, ETM, OLI, MODIS, NOAA/AVHRR, Sentinel series, Gaofen series, SPOT, ALOS, etc), such images have been widely used for desertification monitoring based on long time-series remote sensing data. For example, in[10], the time-series MODIS-normalized difference vegetation index (NDVI) remote sensing data were employed to monitor the trends of aeolian desertification in Horqin Sandy Land from 2000 to 2013. Li et al.[12]obtained the total area of desertified grassland based on spectral mixture analysis (SMA) and DT methods in the Naiman and Ongniud Banners from 1985 to 2013 using Landsat imagery and analyzed the changes in grassland desertification in this region. In[13], SMA was employed to monitor grassland desertification in Tibet, China from 1990 to 2009 using Landsat images. The results demonstrated that the severely desertified grassland area declined from 1990 to 2009, and the grassland desertification area exhibited a gradual reduction during the same period. In [14], the desertification monitoring method was presented based on remote sensing and a geographic information system (GIS), which uses five indices, and the Landsat TM and HJ data were used to analyze the spatial and temporal patterns of the desertification in the Hexi corridor of the Gansu province of northern China from 2000 to 2010. Zhang et al. [15]monitored and assessed the land desertification in the Ebinur Lake region, Xinjiang, China from 1990 to 2010, based on Landsat images and analyzed its cause. The results showed that the desertification occurred rapidly in the study area because of human activity. In[16], the temporal and spatial evolutions of aeolian desertification in the Heihe River Basin from 1975 to 2010 were reconstructed based on visual interpretation methods using multi-temporal Landsat images. The results demonstrated that the expansion of the area of aeolian desertified land (ALD) was gradual from 1975 to 2000 but then decreased rapidly from 2000 to 2010 and that political measures were the principal factors behind the alleviation of desertification. Wang et al. [17] extracted information regarding the dynamic changes of aeolian desertification on the northeast Qinghai–Tibet Plateau from 1987 to 2009 using Landsat TM and Landsat 8 imagery and discussed the spatio-temporal evolution of the landscape patterns of regional ALD. The results show that the dynamics of aeolian desertification in Qinghai Lake basin are mainly determined by climate change, human activity, and management. Zanchetta et al.[18]presented the new Tasselled Cap transform to analyze the desertification changes in Azraq Oasis, Jorand, over 30 years (1984–2013) using Landsat satellites images. The results showed that the proposed method could achieve the expected desertification monitoring. Guo et al. [19] presented a new method based on satellite products and a decision tree (DT) to monitor the spatiotemporal dynamics of desertification in the Ordos Plateau, China from 2000 to 2015 using TM, ETM+, and OLI remote sensing data. In[20], multi-source remote sensing data were used to monitor desertification in Biskra, Ageria. The results showed that the improved method, including radar images, provided excellent results and clearly outperformed other methods based only on optical remote sensing images. According to above, the remote sensing have successfully been used to aeolian desertification monitoring, and the high accuracy of desertification extraction can be obtained. In the feature, the researcher will pay more attention on the desertification monitoring based on multi-source remote sensing and machine learning classification methods. Due to complexity of the WED in karst regions, it is difficult to directly apply the aeolian desertification monitoring methods for WED monitoring in karst regions.

Recently, desertification monitoring on the YGP has attracted the attention of several researchers [21,22]. In[23], the area of karst rocky desertification was extracted in the middle of Guizhou Province from 1974 to 2001 using Landsat images, and the spatiotemporal change pattern of desertification was analyzed. The results showed that the desertification area expanded drastically in the study region over 27 years. In [9], the Landsat 8 OLI data were adapted to monitor karst rocky desertification in Luodian County, Guizhou Province, China in 2015. Wang et al.[24] showed that the Karst Rocky desertification was expanding rapidly in southwestern China. Hu et al. [25] used Landsat TM /ETM images to monitor the temporal and spatial pattern characteristics and regularity of rocky desertification in karst mountainous areas of Guangxi. The DT and fuzzy maximum likelihood (FML) methods were used to extract desertification information, and the results showed that the both methods yielded high-precision results, although the FML method outperformed the DT. Therefore, these desertification monitoring methods can be useful for national desertification surveying.

Recently, rule-based information extraction method has become more popular in remote sensing applications, such as impervious surface extraction, wetland mapping, flood monitoring, and land use/cover classification, so this approach is more transparent to users. Moreover, it allows full evaluation of the data and the passible risks for available categories before defining a particular decision. For example, Xu [26]proposed a rule-based approach to extract imperious surface features from high-spatial-resolution imagery, which provided high overall accuracy in impervious surface extraction. Thus, impervious surfaces were effectively extracted from the soil and water. In[27], a decision rule classification method was developed based upon the hierarchical characteristics of land types and prior knowledge about the geographical locations of wetlands and was used to extract wetlands. The user and producer accuracies of wetland exaction were calculated to be 80.3% and 83.7%, respectively. Ziaei et al. [28]proposed a rule-based system to extract buildings and roads. The results indicated that the proposed method performed satisfactorily, with an overall accuracy with 92.92%, outperforming the support vector machine (SVM) and nearest neighbor methods.

In summary, remote sensing and GISs have been widely employed to monitor desertification and have achieved great success. However, there are relatively few studies on monitoring of the spatiotemporal trends of WED over a long time and on a regional scale, as well as few studies on monitoring of the trends of WED on the YGP using time-series Landsat images. To improve the accuracy of desertification monitoring, this report proposes a multi-indicator rule-based method to extract desertification features. Hence, the objectives of this paper are as follows: (1) to present the multi-indicator rule-based method for WED extraction on the YGP from 1989 to 2016 and (2) to monitor the spatial and temporal trends of WED on the YGP from 1989 to 2016 using Landsat satellite images (Landsat-5 TM, Landsat-7 ETM+, and Landsat-8 OLI). The intention of this report is to provide meaningful information for the government to prevent and control desertification on the YGP.

## Materials and methods

### Study area

**Fig 1:**
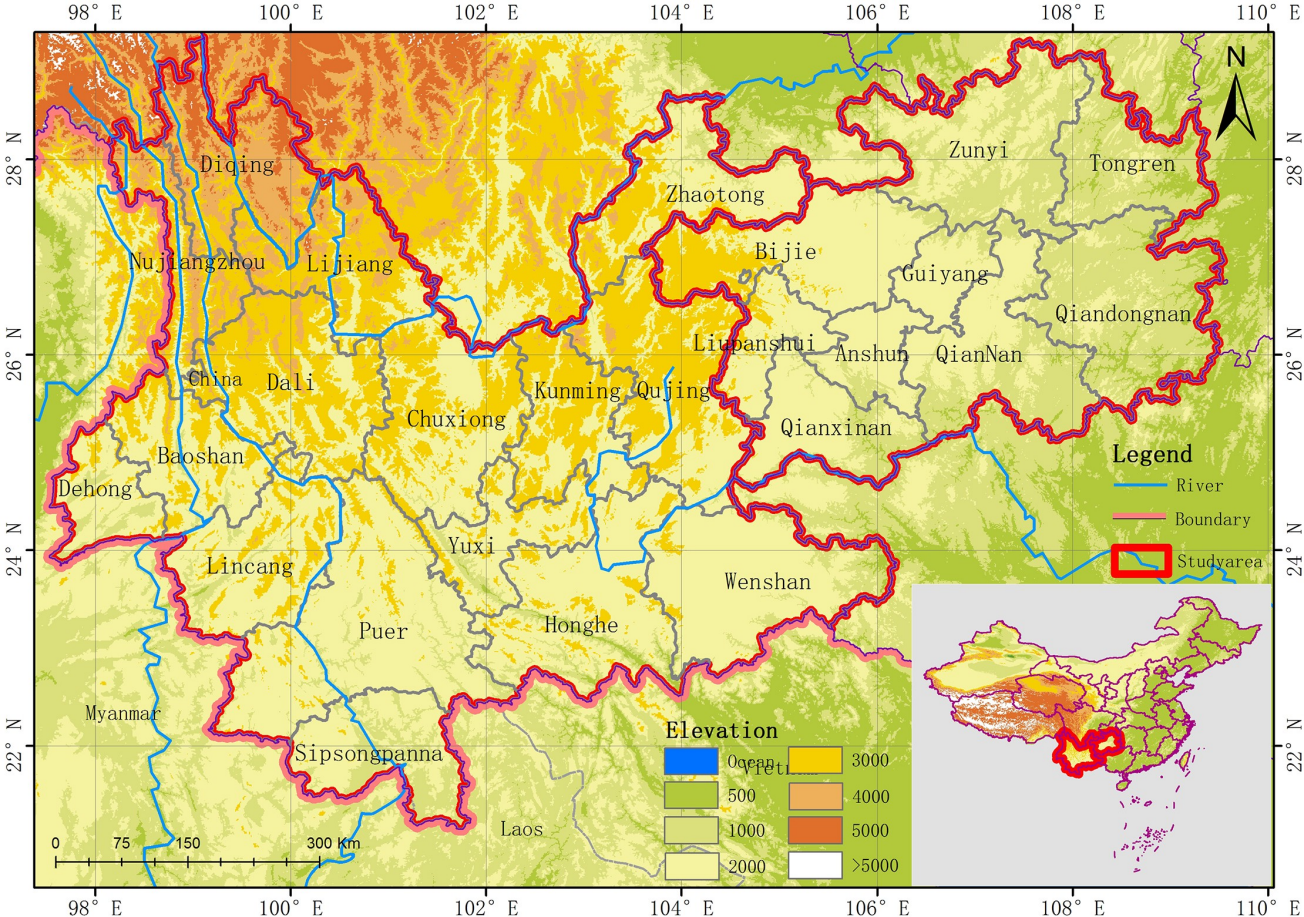
Geographic location of the study area.

The YGP mainly includes Yunnan and Guizhou Provinces, which are located in the southwest of China (**Fig 1**). It lies between 21°8'N and 29°15'N latitude and 97°31'E and 109°15'E longitude, with altitudes between 4000 m and 5000 m, and stretches approximately 123.57 km from east to west and 92.1 km from north to south [29]. The region includes 25 counties, covers 590,200 km^2^, and supports a population of 83.26 million. It has a complex geological environment, which consists of a variety of landforms and terrain elevations. The study area is a subtropical monsoon climate zone, with annual mean precipitation of 600–2000 mm and a mean annual temperature of 5–24°C. Because of the effects of elevation, the vertical climate characteristics in the region are very significant. The YGP is affected by the southwest monsoon, whose rainfall is non-uniform, with a distinct wet season in summer and a dry season in winter [30]. The YGP is the most abundant type of forest vegetation in China, the experimental area is covered by evergreen vegetation[31]. The following basic hydrogeological conditions are found in the experimental area: Guizhou and the eastern part of Yunnan are dominated by carbonate lithology, however, central Guizhou is dominated by clastic rocks, and western Yunnan is dominated by metamorphic rocks[32] The lithology determines the poor water retention function in the experimental area. In summary, the geological conditions on the GYP are complex and the natural environment is vulnerability. Therefore, it is important to study the water-erosion desertification in the Yunnan and Guizhou provinces.

### Data collection and preprocessing

#### Data collection

*Landsat satellite images*. The Landsat series satellite data are of moderate spatial resolution (30 m) and contain the longest temporal record of space-based Earth surface observations, offering a unique opportunity to observe anthropogenic and natural changes at local to global scales[9,12,33]. To achieve the research objective of monitoring the trend of WED on the YGP over the past 27 years, we selected Landsat thematic mapper images acquired in 1989, Landsat thematic mapper images acquired from 1992 to 2010, Landsat ETM Plus (ETM+7) images acquired in 2001, and Landsat OLI images acquired from 2013 to 2017. The long time-series Landsat images of the region on cloudless days or days with cloud cover of no more than 5% were selected and downloaded from the United States Geological Survey website (http://glovis.usgs.gov) for free. The remote sensing imagery datasets used in this study are shown in **Table 1**.

**Table 1:** Landsat dataset used in this study.

Item	Image acquisition data (year/month/day)	Number of scenes	Orbit number	Data source
1	1989/01/11–1989/10/20	46	125/40 125/41 125/42 126/40 126/41 126/42 126/43 127/40 127/41 127/42 127/43 127/44 128/40 128/41 128/42 128/43 128/44 129/40 129/41 129/42 129/43 129/44 129/45 130/41 130/42 130/43 130/44 130/45 131/41 131/42 131/43 131/44 131/45 132/40 132/41 132/42 132/43 132/44	Landsat4 TM Landsat5 TM
2	1992/01/05–1992/12/08	43		Landsat5 TM
3	1995/01/11–1995/12/20	42		Landsat5 TM
4	1998/01/03–1998/12/28	43		Landsat5 TM
5	2001/01/011–2001/12/15	44		Landsat5 TM Landsat7 ETM+
6	2004/01/31–2005/01/31	39		Landsat5 TM
7	2007/01/09–2007/09/20	44		Landsat5 TM
8	2009/12/12–2010/12/29	42		Landsat5 TM
9	2013/04/15–2013/12/05	39		Landsat8 OLI
10	2016/8/29–2017/4/29	44		Landsat8 OLI

*Digital Elevation Model (DEM)*. DEM data were needed in this study and were used to extract the slope and gully density indicators. High-resolution global DEMs (GDEMs) have been generated from the Aster stereoscopic datasets, which are publicly available for free. At present, GDEMs cover the land surfaces between 83°N and 83°S with a spatial resolution of 1ʺ (approximately 30 m) and coverage of about 99% of the land areas on Earth. The GDEMs in the YGP were selected and downloaded from the Geospatial Data Cloud website (http://www.gscloud.cn).

*Training and validation samples*. In this study, the validation samples were needed to assess the desertification extraction accuracies of the different methods, and the training samples were used in the Support Vector Machine (SVM) and Muti-information Decision Tree (MIDT) methods. WED can be divided into three classes (slight, SLD; moderate, MD; and severe, SD). The sample plots corresponding to different WED classes were selected through manual interpretation using high-spatial-resolution images from Google Earth. Half of the samples were randomly selected for training, and the remaining samples were used for validation, as shown in **Table 2**.

**Table 2:** Numbers of pixels used for training and validation.

Degree of desertification	Land surface characteristics	Number of samples
Mild	The mild desertification areas in the image are blue-gray or yellow-gray, and vegetation and cultivated land are covered by mixed rocks	3103
oderate	The moderate desertification areas in the image are mainly dark gray or bright yellow, occur on the slope surface, and suffer from water erosion. Rock bare and bare soil occur throughout the area, but the color in the image is slightly darker than mild desertification	3115
Severe	The severe desertification areas in the image are gray or yellowish white, mostly occur on the slope surface, and suffer from special water erosion. Rock bare and bare soil occur throughout the area.	3110

### Preprocessing of remote sensing data

The pre-processing of the long time-series images mainly included atmospheric correction, geometric correction, and image registration [6]. Firstly, all of the images were atmospherically corrected using the Fast Line-of-sight Atmospheric Analysis of Spectral Hypercube (FLAASH) module of ENVI 5.3. Next, geometric correction was performed on the Landsat images in 2016 by selecting ground control points from topographic maps with rectification errors less than one pixel. Finally, the corrected images were used to rectify other images by performing image-to-image registration in which the second-order polynomial model was applied, where the total root-mean-square error was less than 0.5 pixel.

### Rule-based method with multiple indictors

Recently, numerous other indictors (such as the ratio vegetation index, difference vegetation index, NDVI, fractional vegetation coverage (FVC), modified vegetation index, modified soil-adjusted vegetation index, net primary productivity, temperature-vegetation drought index, land surface temperature, surface albedo, and bare soil index) have been used for desertification monitoring and assessment. However, multiple indicators, including topographical indictors, were not considered in the previous methods. Because of taking advantage of the rule-based method and the differences of multiple indictors in different degrees of desertification. Thus, this paper proposes a multi-indicator rule-based method, which considers the FVC, fraction of underlying coverage (FUC), slope, and gully density.

#### Calculation of monitoring indicators

In this study, three types of indicators were extracted, including FVC, which is a kind of vegetation index; FUC, which indicates whether the soil under vegetation is not rock or bare soil; and the topographical indictors slope and gully density, which indirectly represent the degree of lateral erosion and down-erosion by surface water flow, respectively.

*FVC*. which is defined as the percentage of the vertical projected area of vegetation to the total ground area, is an important parameter for characterizing the land surface vegetation conditions[34]. A dense vegetation mosaic-pixel model is the most effective and widely used to retrieve vegetation cover. The FVC can be calculated by applying the following equation (Eq 1)

$$FVC=\frac{NDVI-NDVI_{soil}}{NDVI_{veg}-NDVI_{soil}}$$ (Eq 1)

where NDVI is the vegetation index value of one pixel; *NDVI*_*soil*_ is the *NDVI* of a pure soil pixel, which is extracted from the mean *NDVI* of the bare soil pixels identified in the classification results; and *NDVI*_*veg*_ is the value of a pure green vegetation pixel, which is obtained from the histogram of the *NDVI* in the whole study area. The *FVI* in the study area was extracted using the Eq 1 from 1989 to 2016.

*FUC*. Currently, there is no effective method of estimating bedrock exposure rate using remote sensing techniques[35]. According to the true situation in the southwest, the surface is covered by bare soil and rock. In [36], the normalized difference underlying index (NDUI) was presented based on the spectral difference between bare soil and rock, which are well correlated with the underlying surface exposure rate. The NDUI is calculated from in the near-infrared (NIR) and second shortwave-infrared (SWIR_2_) bands of Landsat multi-spectral data as follows (Eq 2).

$$NDUI=\frac{\rho_{\mathrm{NIR}}-\rho_{SWIR2}}{\rho_{NIR}-\rho_{SWIR2}}$$ (Eq 2)

Where *ρ*_*NIR*_ and *ρ*_*SWIR*2_ are the spectral reflectance in the *NIR* and *SWIR*_2_, respectively.

We presented the *FUC* based on the *FVC* principle, which is more able to improve the difference between bare soil and rock. The *FUC* can be obtained by combining the *NDUI* and dimidiate pixel model as follows (Eq 3):

$$FUC=\frac{NDUI-NDUI_{veg}}{NDUI_{soil}-NDUI_{veg}}$$ (Eq 3)

Where *NDUI* is the *NDUI* of an individual pixel and *NDUI*_*soil*_ and *NDUI*_*veg*_ are the NDUIs in the pure soil and vegetation pixels, respectively.

The FUC in the study area was extracted using the Eq 3 from 1989 to 2016.

**Fig 2:**
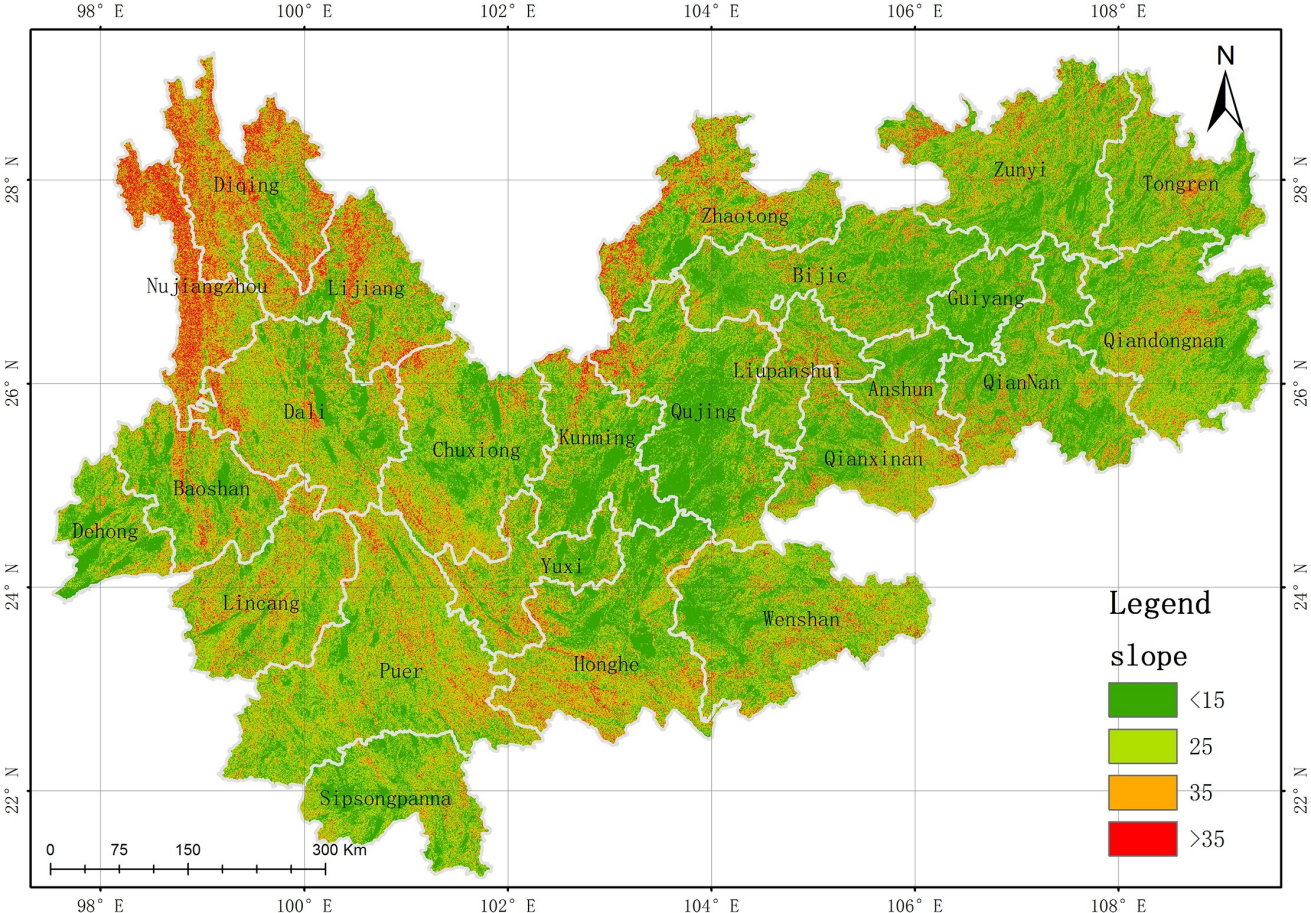
Slope map of study area. (USGS/NASA Landsat).

**Fig 3:**
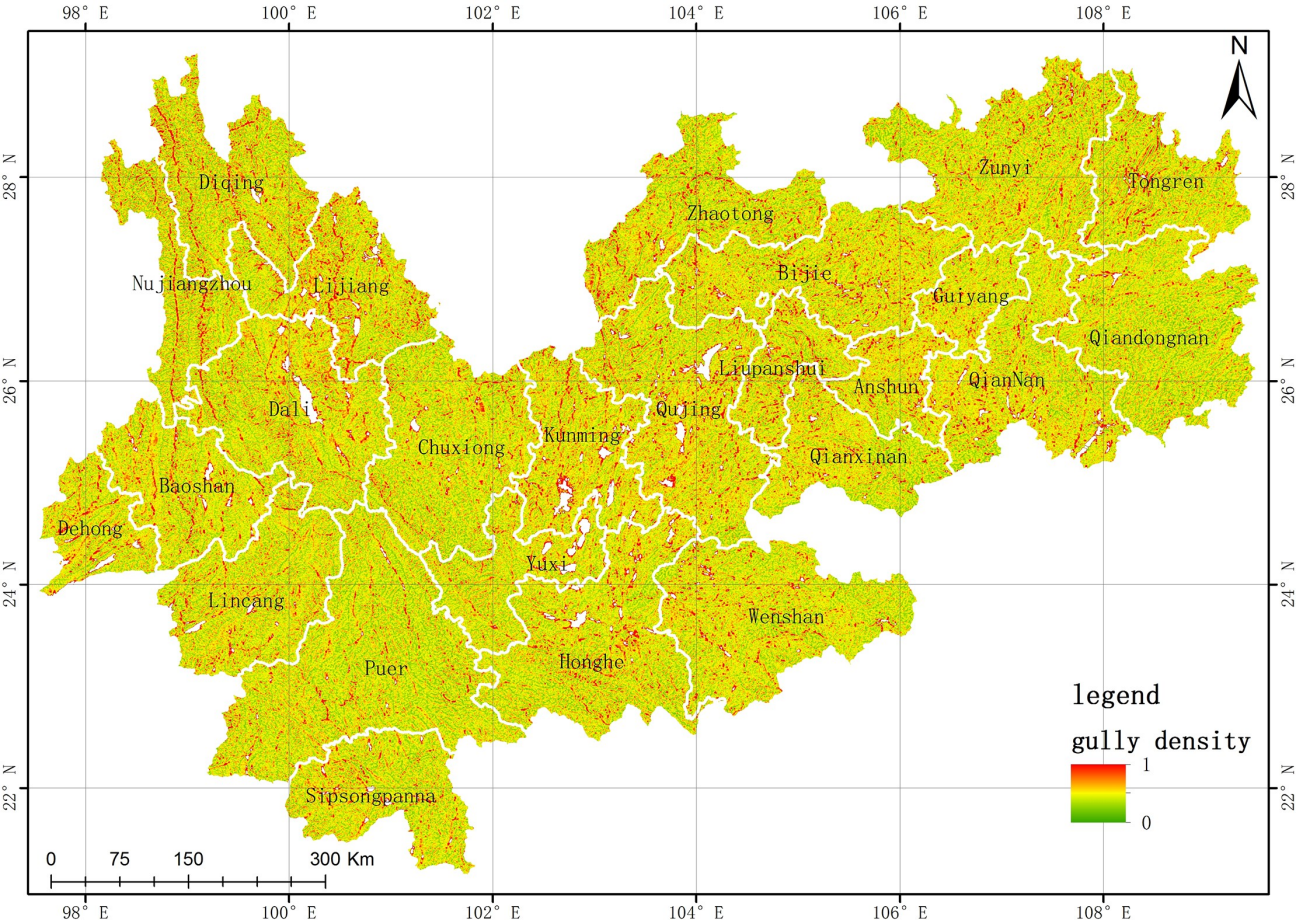
Gully density map of study area. (USGS/NASA Landsat).

*Slope and gully density*. The slope and gully density are two topographical indictors. The slope represents the degree of lateral erosion by surface water flow[34], and the gully density describes the degree of ground cutting and down-erosion by surface water flow[33,35]. In this study, the slope and gully density were obtained from 30 m GDEM data by using the spatial analysis module in ArcGIS software (Fig 2 and Fig 3, respectively)

#### Rule-based method

The rule formation involves learning the relationships between the indicators and classes to construct a rule-based tree model [26]. Rule-based information extraction is a classification procedure in which a data set is iteratively segmented into smaller subdivisions based on rule sets. The rule-based classifier in this study works by evaluating the values of the four indicators in individual pixels and classifying each pixel into different classes according to the thresholds of each rule. The rules used in this study to map the water-erosion desertification in the YGP using Landsat images were analyzed and obtained based on the signature differences between different degrees of desertification within the four indictors.

**Fig 4:**
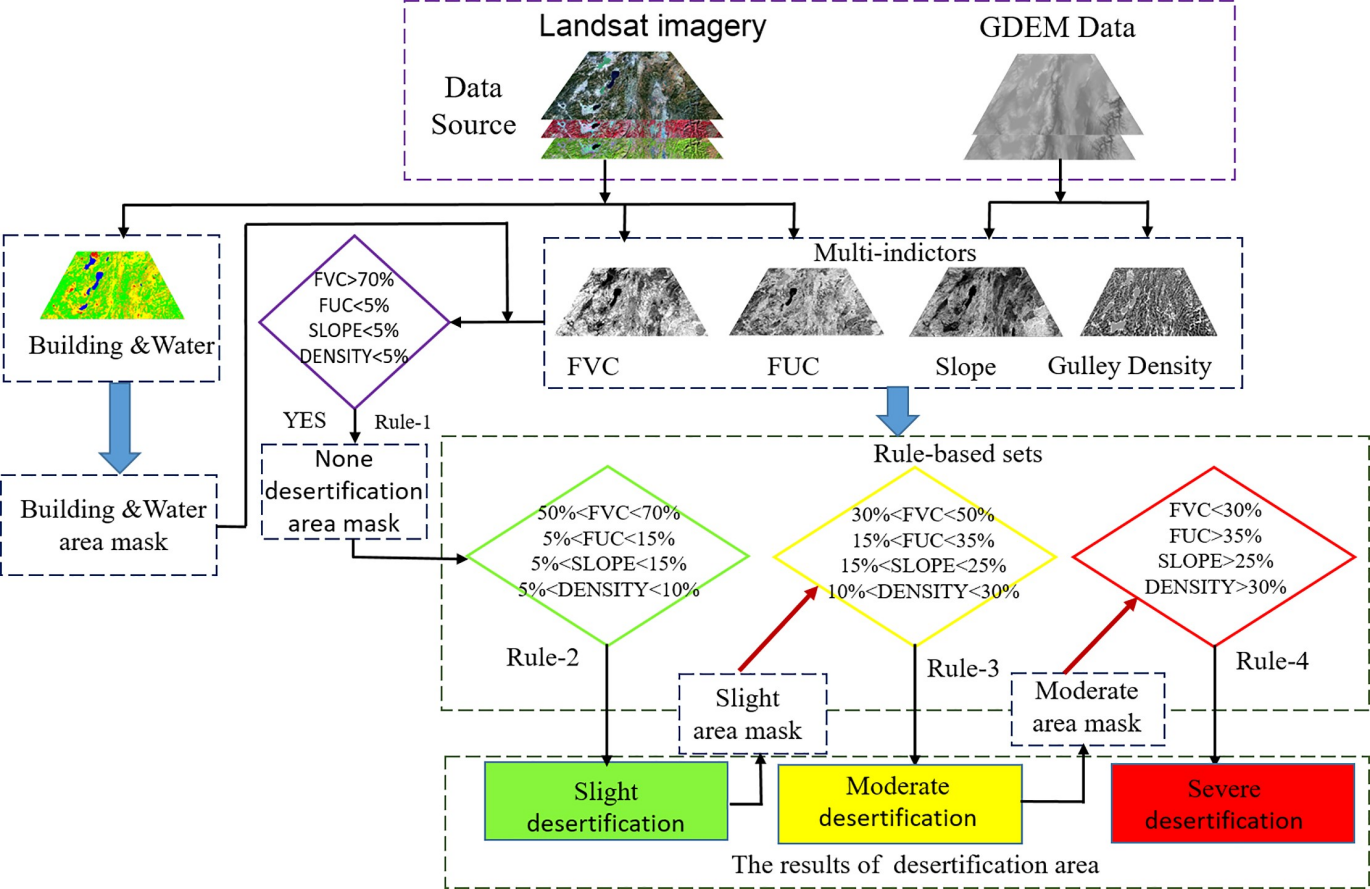
Rules and thresholds used to extract desertification from the Landsat images.

Four rules were established based on the four indictors and are expressed in the rule-based model (**Fig 4**). The rule sets were constructed as follows. (1) Firstly, areas without desertification were masked by the land use/cover classification data and rule 1, where the FVC is greater than 70%, the FUC is less than 5%, the slope is less than 5%, and the gully density is less than 5%. (2) Secondly, based on the first step in which areas without desertification are masked, the SLD areas are classified by rule 2, where the FVC is less than 70% but greater than 50%, the FUC is less than 15% but greater than 5%, the slope is less than 15% but greater than 5%, and the gully density is less than 10% but greater than 5%. (3) Thirdly, according to the results of the second step, the areas with SLD are masked. The MD areas are classified by rule 3, where the FVC is less than 50% but greater than 30%, the FUC is less than 35% but greater than 15%, the slope is less than 25% but greater than 15%, and the gully density is less than 30% but greater than 10%. (4) Fourthly, according to the results of the third step, the MD areas are masked. The SD areas are classified by rule 4, where the FVC is less than 30%, the FUC is greater than 35%, the slope is greater than 25%, and the gully density is greater than 30%. The thresholds of the four indictors in the rule sets were obtained by calculating the average values of the training samples corresponding to each degree of desertification.

### Driving factors

The WED desertification is a comprehensive process related to many factors such as ecology, geography, climate, and humanities. It is closed relationship with not only natural environment but also human activity, social and economic development, and the regional government [37]. Therefore, the complex nature effects, human activities, and social economy should be considered for the WED desertification on the YGP. In the previous researches, the foundational driving factors of the WED desertification mainly include climate, land cover, and social factors [36]. In this paper, we have selected the precipitation and temperature as climatic factors, and water area, vegetation area, bare soil area, cultivated area, and construction area as land cover factors, and select GDP and population as society. The correlation analysis method was adopted to analyze the importance among the above factors. Finally, the precipitation, vegetation area, and GDP are considered as important driving factors in this paper.

## Results and discussion

### Comparison of classification methods

The method proposed in this paper is a rule-based method considering four indictors (FVC, FUC, slope, and gully density), called VUSG method. By using different indictor combinations, five other classification methods were designed using the same rule base and thresholds as in the proposed method. Specifically, the alternative classification methods were developed using the FVC, slope, and gully density (VSG method); FUC, slope, and gully density (USG method); FVC and FUC (VU method); FVC only (FVC method), and FUC only (FUC method). The DT method was selected using four indictors and the seven bands as input features in the experiments, and the SVM was selected using the features of the seven bands as input features. The same categories, training sets, and accuracy assessment samples were used in the VUG, VUS, USG, VSG, VU, DT, and SVM methods as in the proposed method to ensure compatibility.

**Fig 5:**
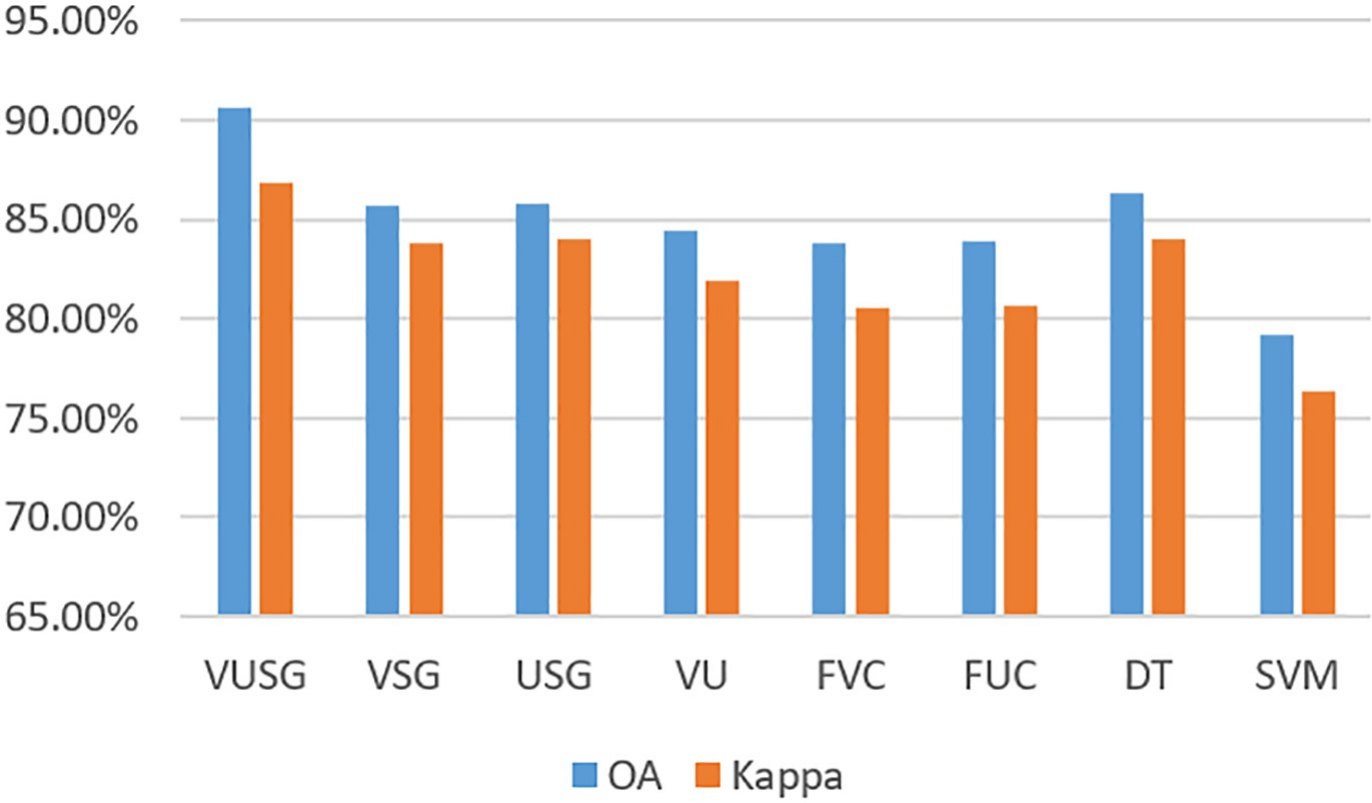
Histogram of classification accuracies of different methods.

The eight classification methods were utilized to extract the water-erosion desertification on the YGP in 2010, and the Overall Accuracy (OA) and Kappa Coefficient (kappa) were employed to evaluate the classification accuracies of the different methods, which are shown in Table 3 and Fig 5.

**Table 3:** Classification accuracies of different methods.

Methods	VUSG	VSG	USG	VU	FVC	FUC	DT	SVM
OA	90.61%	85.73%	85.82%	84.42%	83.85%	83.88%	86.34%	79.21%
Kappa	86.85%	83.82%	83.96%	81.95%	80.59%	80.67%	84.02%	76.33%

According to the experimental results, the VUSG method obtained the highest classification accuracy in terms of OA and kappa, which were 90.61% and 86.85%, respectively. Among the rule-based classification methods, the VSG and USG methods, which are based on topographical indictors (slope and gully density), have higher accuracies than the VU, FVC, and FUC methods, which do not include topographical indictors. The multi-indicator VUSG, VSG, USG, and VU methods provided better accuracy than the FVC and FUC methods based on single indictors. Among the machine learning methods, the DT based on multiple indictors and band features outperforms the SVM method based on band features in terms of OA and kappa by 7.13% and 7.69%, respectively. Meanwhile, the DT method yielded more accurate classification results than the VSG, USG, VU, FVC, and FUC methods. In summary, the WED classification accuracy can be improved by combining the FCV and FUC, which is important for WED extraction. These indictors can improve the differences between degrees of desertification.

### Spatial-temporal changes analysis of WED from 1989 to 2016

**Fig 6:**
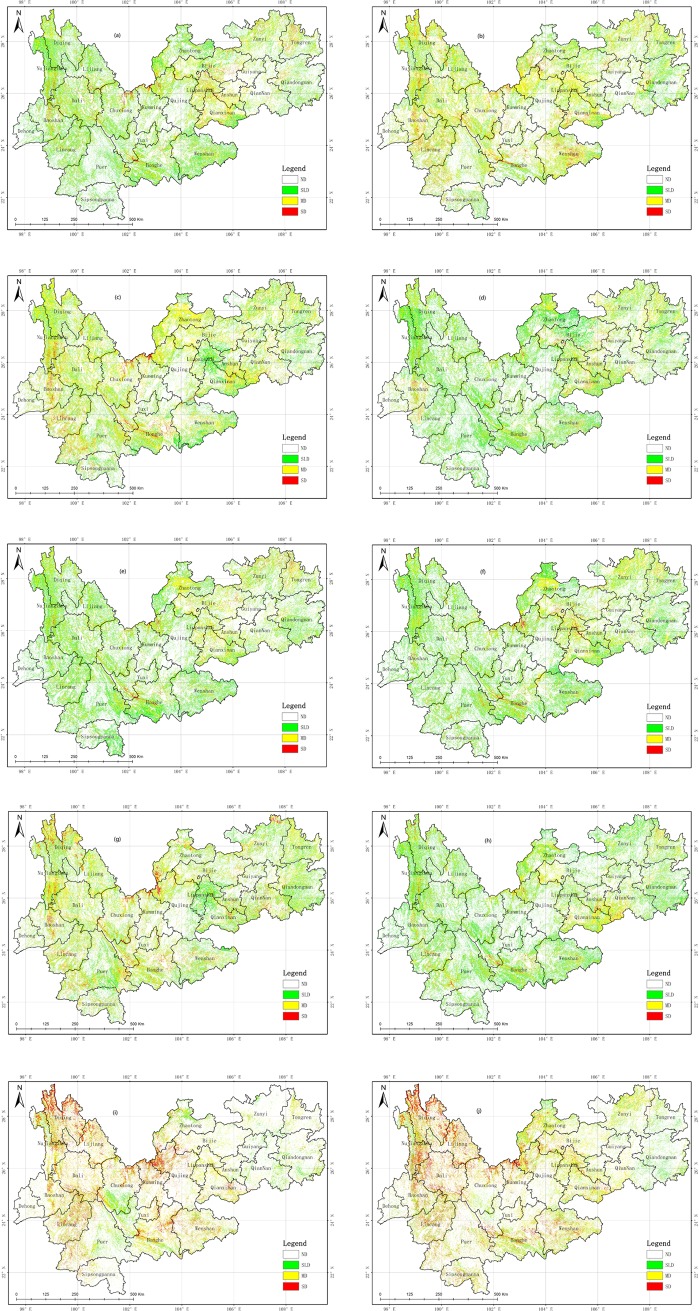
Spatial distribution of desertification in study area during (a) 1989; (b) 1992; (c) 1995; (d) 1998; (e) 2001; (f) 2004; (g) 2007; (h) 2010; (i) 2013; and (j) 2016. (USGS/NASA Landsat).

The WED is classified as three types: SLD, MD, and SD. The thematic maps of the WED in the YGP over 10 periods from 1989 to 2016 were obtained based on the method proposed in this paper and are shown in **Fig 6**. According to the thematic maps, we determined that the WED regions are mainly distributed in the Dian–Gui–Qian Junction region, which contains southwestern Yunnan, southern Anshun, and Liupanshui in Guizhou; Wenshan and Honghe in central Yunnan; the border region in western Yunnan, which contains Nujiang, Diqing, Lijiang, Dali, Baoshan, Puer, and the Wumengshan region, which consists of Chuxiong, Kunming, Qujing and Zhaotong in Yunnan, and Bijie in Guizhou. The **Table 4** shows the area of each degree of WED and its proportion of the total area in each of the 10 periods from 1989 to 2016. The statistical results indicate the following. (1) The total WED area increased from 17.80×10^4^ km^2^ in 1989 to 20.50×10^4^ km^2^ in 2016 with an annual growth rate (AGR) of 0.56% (2) The monitoring results indicate an increase in SLD from 89,617.09 km^2^ in 1989 to 100,976.47 km^2^ in 2016 with and AGR of 0.48%, an increase in MD from 80,276.65 km^2^ in 1989 to 90,768.39 km^2^ in 2016 with an AGR of 0.50%, and an increase in SD from 8149.3 km^2^ in 1989 to 13,220.16 km^2^ in 2016 with an AGR of 2.39%. (3) The total areas of the different degrees of WED shrank in 1992, 1998, 2001, and 2004, and the total WED area increased slowly from 17.80×10^4^ km^2^ in 1989 to 17.98×10^4^ km^2^ in 2010 with an AGR of 0.05%. The SLD area decreased from 89,617.09 km^2^ in 1989 to 89,258.53 km^2^ in 2010 with an AGR of 0.02%, the MD area increased from 80,276.65 km^2^ in 1989 to 82,191.31 km^2^ in 2010 with an AGR of 0.11%, and the SD area increased form 8149.3 km^2^ in 1989 to 8380.3 km^2^ in 2010 with an AGR of 0.26%. However, the WED rapidly expanded in the YGP from 2010 to 2013, increasing from 17.98×10^4^ km^2^ to 20.28×10^4^ km^2^ with an AGR of 4.26%. Meanwhile, the SLD area increased from 89,258.53 km^2^ in 2010 to 98,496.56 km^2^ in 2013 with an AGR of 3.45%, the MD area increased from 82,191.31 km^2^ in 2010 to 91,314.09 km^2^ in 2013 with an AGR of 3.70%, and the SD area increased from 8380.30 km^2^ in 2010 to 13,029.22 km^2^ in 2013 with an AGR of 18.49%, due to the severe drought in the YGP in 2010. However, the WED remained stable in the YGP from 2013 to 2016. The total WED area increased from 20.28×10^4^ km^2^ in 2013 to 20.50×10^4^ km^2^ in 2016 with an AGR of 0.36%, the SLD area increased from 98,496.56 km^2^ in 2013 to 100,976.47 km^2^ in 2016 with an AGR of 0.84%, the MD area decreased from 91,314.09 km^2^ in 2013 to 90,768.39 km^2^ in 2016 with an AGR of 0.20%, and the SD area increased from 13,029.22 km^2^ in 2013 to 13,220.16 km^2^ in 2016 with an AGR of 0.49%. In summary, the WED expansion on the YGP can be divided into three stages as follows: a slow increase from 1989 to 2010, rapid expansion from 2010 to 2013, and a slight increase from 2013 to 2016.

**Table 4:** Areas and proportions of different degrees of WED from 1989 to 2016 (area: km2; proportion: %).

	SD		MD		SLD		ND	
	Area	Proportion	Area	Proportion	Area	Proportion	Area	Proportion
1989	8149.3	1.49	80,276.65	14.64	89,617.09	16.34	370,284.05	67.53
1992	7943.03	1.45	84,364.41	15.39	87,260.37	15.91	368,759.28	67.25
1995	8325.02	1.52	83,297.90	15.19	90,337.20	16.48	366,366.97	66.82
1998	8091.85	1.48	79,397.26	14.48	91,313.3	16.65	369,524.68	67.39
2001	7967.72	1.45	81,913.78	14.94	87,906.61	16.03	370,538.98	67.58
2004	8041.98	1.47	81,277.57	14.82	90,980.30	16.59	368,027.25	67.12
2007	8990.21	1.64	82,737.08	15.09	91,562.08	16.70	365,037.73	66.57
2010	8380.30	1.53	82,191.31	14.99	89,258.53	16.28	368,496.95	67.20
2013	13,029.22	2.38	91,314.09	16.65	98,496.56	17.96	345,487.22	63.01
2016	13,220.16	2.41	90,768.39	16.55	100,976.47	18.42	340,362.08	62.07

### Dynamic changes and transfers between different degrees of WED from 1989 to 2016

**Fig 7:**
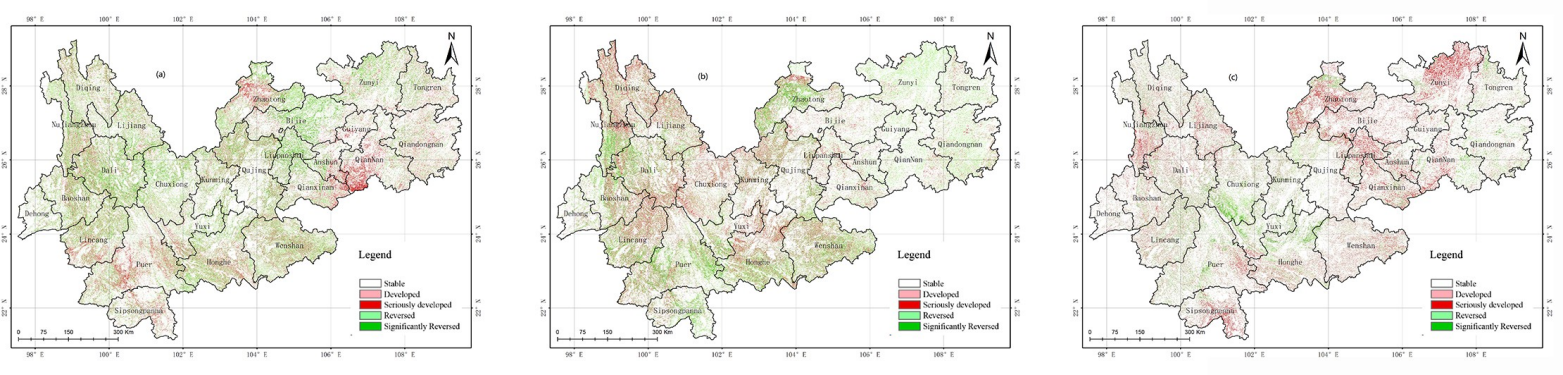
Trends of desertification changes from 1989 to 2010, 2010 to 2013, and 2013 to 2016. (USGS/NASA Landsat).

**Fig 8:**
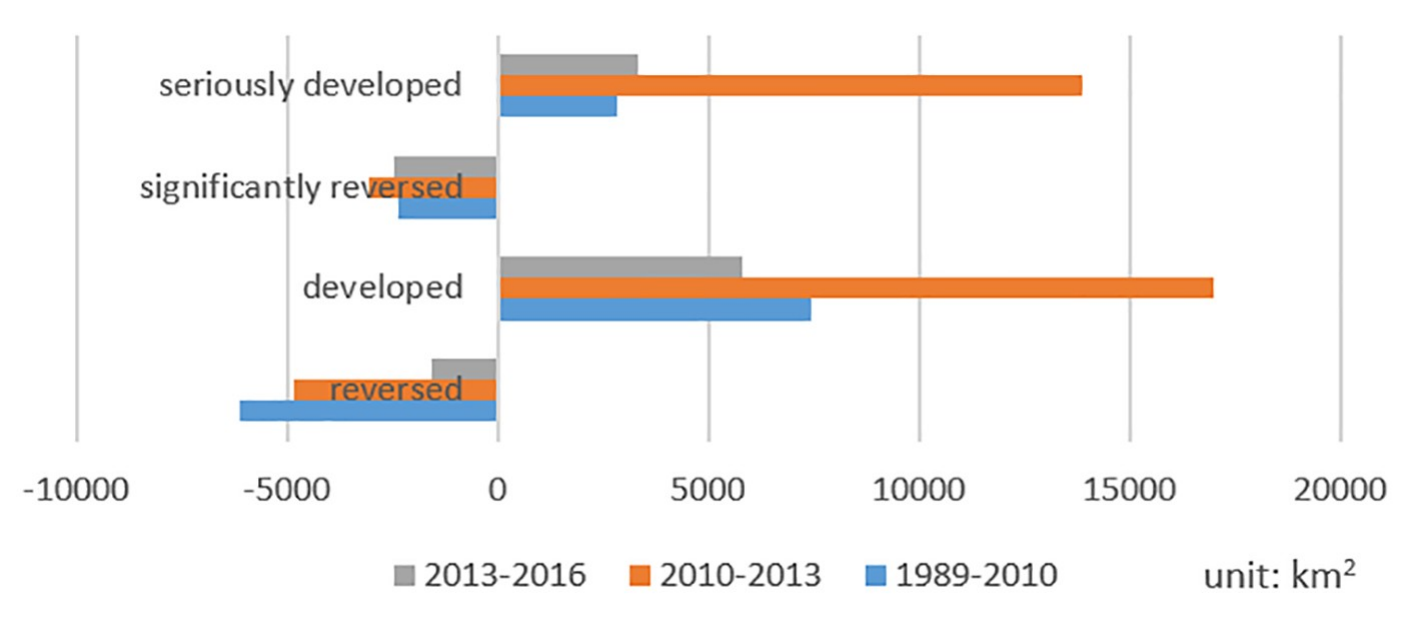
Trends of area changes between different degrees of WED in three periods.

**Fig 9:**
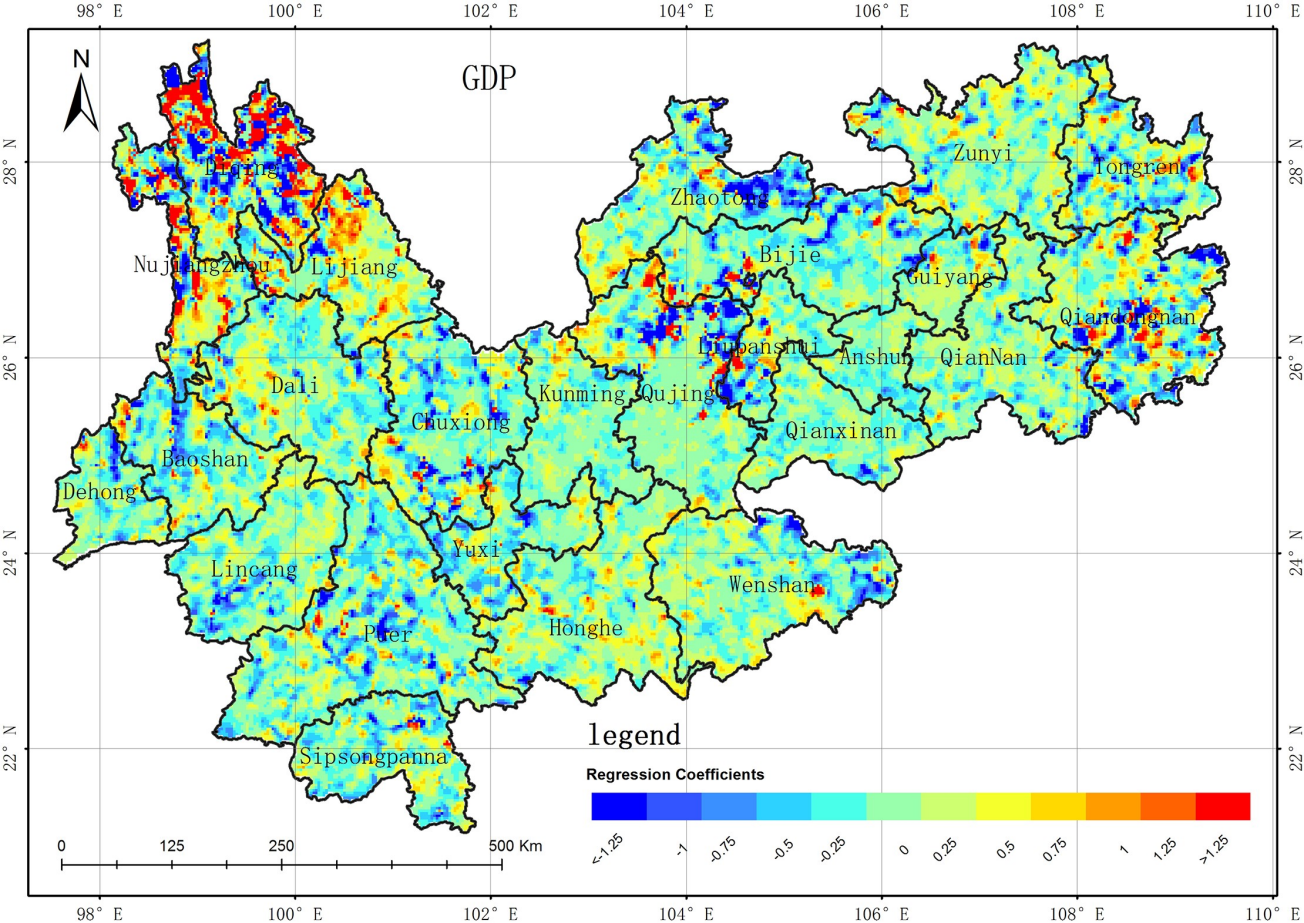
Spatial distribution of GWR regression coefficient between GDP and WED in the YGP. (USGS/NASA Landsat).

Based on the above spatiotemporal change analysis of WED on the YGP from 1989 to 2016, the dynamic changes and transfers between different degrees of WED in the three periods (1989–2010, 2010–2013, and 2013–2016) were analyzed, which are shown in **Fig 7**. The results were classified into five categories as follows: an increase in the degree of desertification was designated as “developed” (e.g., a change from SLD to MD); a cross-level increase in the degree of desertification was defined as “seriously developed” (e.g., a change from SLD to SD); a decrease was designated as “reversed” (e.g., a change from MD to SLD); a cross-level decrease was regarded as “significantly reversed” (e.g., a change from SD to SLD), and an area without change in the degree of desertification was defined as “stable.” According to **Fig 8** and **Table 5**, the statistical results are as follows. (1) From 1989 to 2010, the regions of developed and seriously developed desertification mainly occurred in southwestern Yunnan (such as in Licang, Puer, and Sipsongpanna), southern Guizhou (such as in Anshun, Qiannan, and Qianxinan), and Zhaotong, with areas of 7429.30 km^2^ and 2833.90 km^2^, respectively; the reversed and significantly reversed regions were distributed in the Dian–Gui–Qian region, border region in western Yunnan, and central Yunnan, with areas of 6140.01 km^2^ and 2336.09 km^2^, respectively. (2) From 2010 to 2013, the regions of developed and seriously developed desertification were mainly distributed in central Yunnan (such as in Kunming, Qujing, and Chuxiong), the border region in western Yunnan (such as in Diqing, Nu jiangzhou, and Lijiang), and the Dian–Gui–Qian region, with areas of 16,997.96 km^2^ and 13,889.32 km^2^, respectively. In the same period, reversed or significantly reversed regions mainly occurred in Guizhou and southwestern Yunan, with areas of 4813.31 km^2^ and 3064.24 km^2^ respectively. (3) From 2013 to 2016, the regions of developed and seriously developed desertification were mainly distributed in the Dian–Gui–Qian region, Zunyi, and central Guizhou (such as in Bijie, Liu Panshui and Anshun), with areas of 5810.14 km^2^ and 3306.19 km^2^, respectively. In the same period, the developed and seriously developed desertification regions reversed and significantly occurred in central Yunnan (such as Kunming, Chuxiong, and Yuxi) and eastern Guizhou, with areas of 1555.15 km^2^ and 2436.04 km^2^, respectively. Based on Fig 9, the following conclusions were obtained. The dynamic desertification changes in the first period (from 1989 to 2010) were obviously slighter than those in the other two periods (from 2010 to 2013 and from 2013 to 2016), and from 2010 to 2013, the desertification areas expanded dramatically compared with the period from 2013 to 2016. (4) In Tongling and Zunyi interior, northeastern Guizhou, the WED desertification shows downward trends during the 2010–2016 period. According to the planning of the WED desertification control projects in the GuiZhou province, it was found that some desertification control projects were implemented on the above areas the past several years. So we obtained the conclusion that the government desertification control projects can improve the WED desertification on the GYP.

**Table 5:** Dynamic changes between different degrees of WED in three periods (area: km2).

	Reversed	Developed	Significantly reversed	Seriously developed	Total
1989–2010	6140.01	7429.30	2336.09	2833.90	1787.10
2010–2013	4813.31	16,997.96	3064.24	13,889.32	23,009.73
2013–2016	1555.15	5810.14	2436.04	3306.19	5125.14

### Driving factor analysis

**Fig 10:**
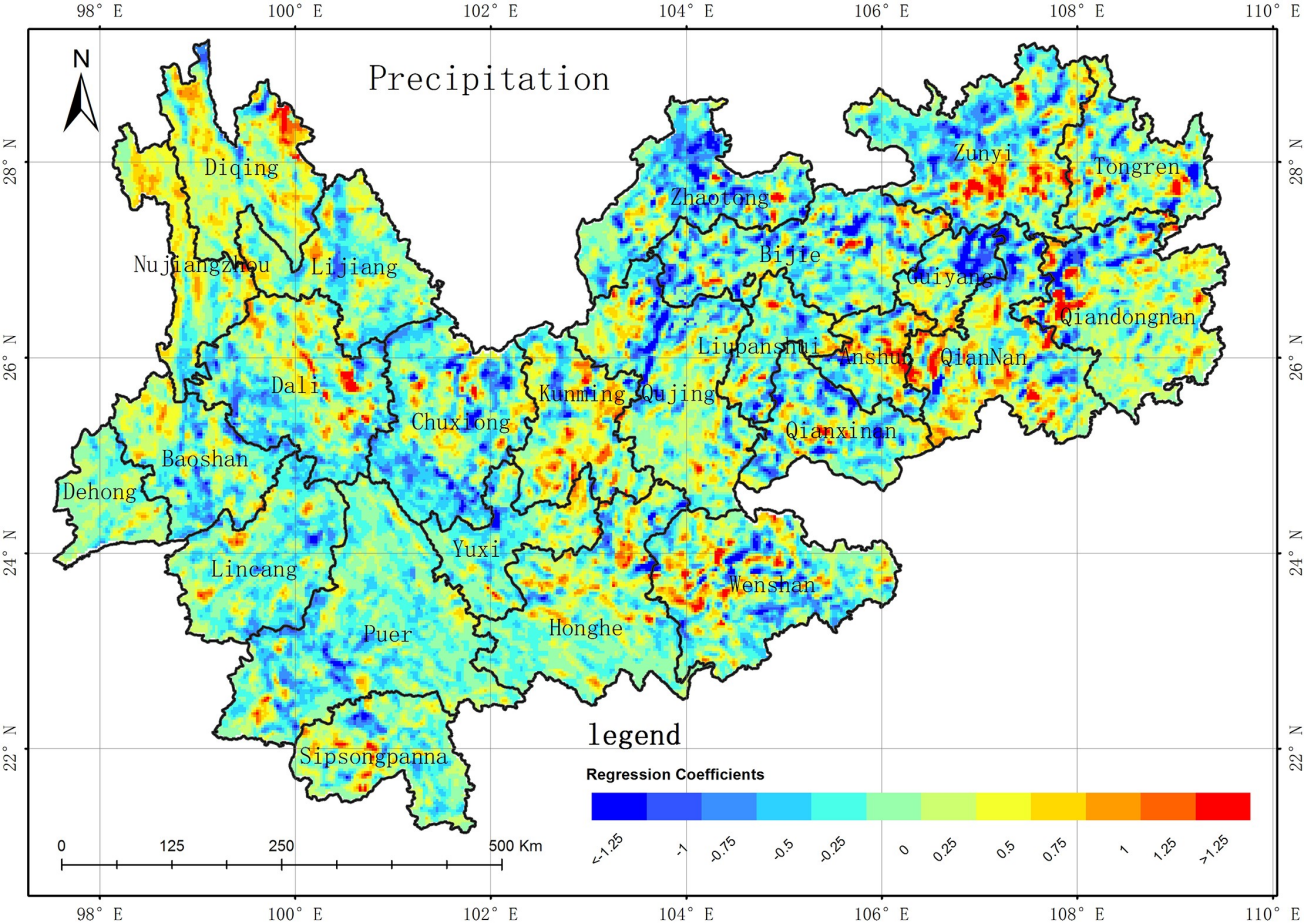
Spatial distribution of GWR regression coefficient between precipitation and WED in the YGP. (USGS/NASA Landsat).

**Fig 11:**
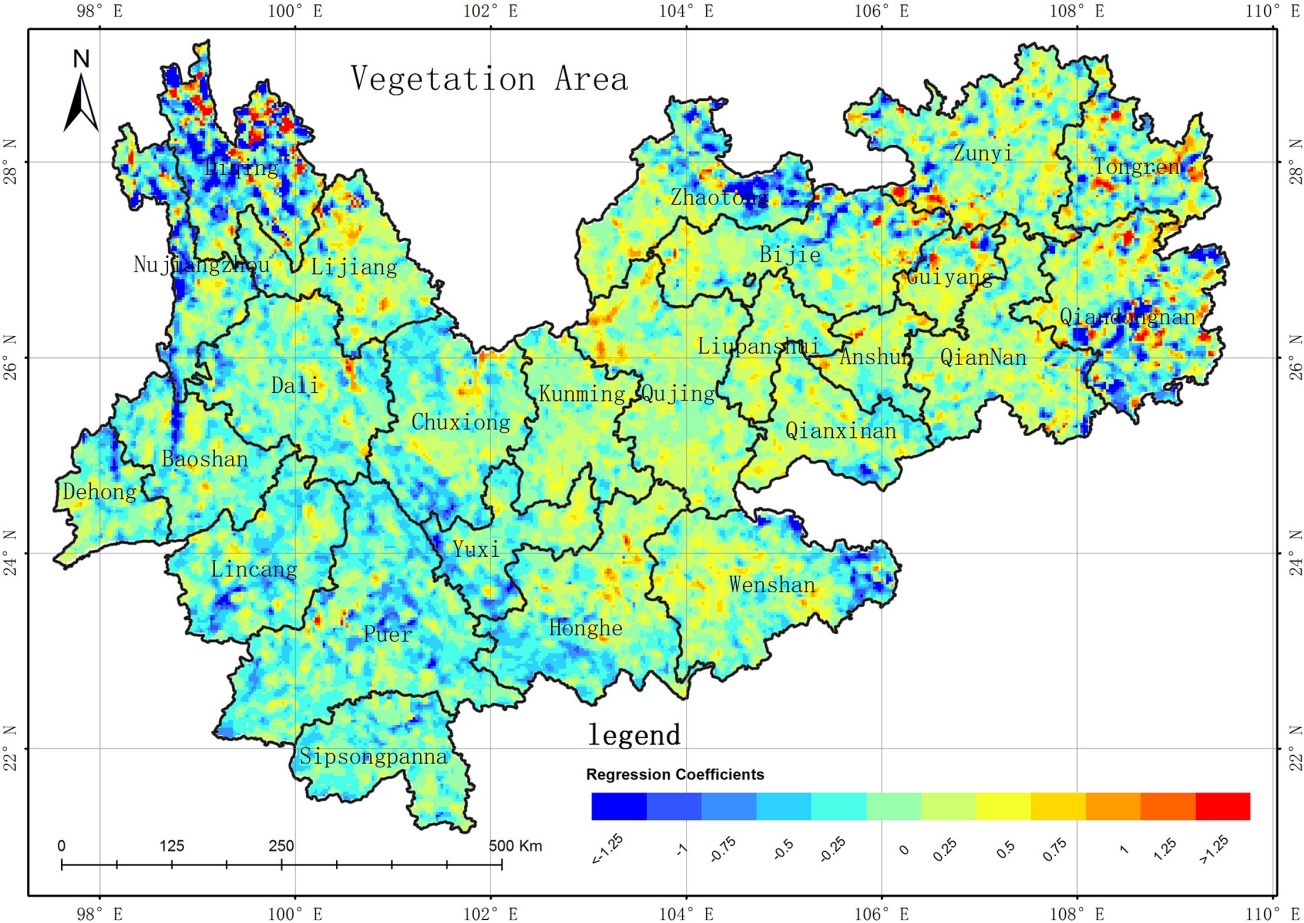
Spatial distribution of GWR regression coefficient between vegetation area and WED in the YGP. (USGS/NASA Landsat).

Many factors can contribute to the desertification changes on the YGP, including climate (precipitation and temperature), socioeconomic changes (population and gross domestic product (GDP)), and human activity (land use/cover changes among vegetation, soil, impervious surface, and cultivated land). The correlation analysis method[36] was employed to obtain the key factors influencing desertification, which were determined to be the precipitation, vegetation area, and GDP according spatial autocorrelation result. Due to spatial differences on a large scale, it is difficult to obtain better correlations between desertification and these factors using a linear regression model[38,39]. Recently, geographically weighted regression (GWR), which can be used to model spatial variations in the relationships between dependent and independent variables, has attracted increasing research attention[40,41]. Thus, GWR was introduced to analyze the correlation between desertification and the aforementioned factors in this study[42,43]. Firstly, the regression coefficients between desertification and each factor were obtained using the GWR from 1989 to 2016; secondly, the average values of the regression coefficients in the 10 periods (from 1989 to 2016) were computed for each factor, whose spatial distributions are shown in Figs 9–11. The conclusions can be summarized as follows. (1) The regression coefficients of each factor contain both positive and negative values. The results show that there are significant differences in the correlations between the factors and WED in terms of spatial distribution, degree of influence, and positive and negative effects. (2) The GWR regression coefficient between GDP and desertification area is negatively correlated (**Fig 9**), but there are still some areas where the GWR regression coefficient is positively correlated. In the 10 series results, the areas of negative correlation (regression coefficient -1~0) are mainly distributed in Diqing, Nujiang, Lijiang, Baoshan, Dehong, Linyi and Pu'er. Xishuangbanna, the southwestern part of Chuxiong, the eastern part of Wenshan City, the southeastern part of Wendong City, the southeastern part of Zhaodong City, and the northeastern part of Zhaotong City. These are all poverty-stricken areas of the YGP. The domestic economy is based on agriculture and relies heavily on poverty alleviation and industrial development. The development of agriculture effectively reduces the pressure on the land while controlling desertification. Therefore, the GDP of these areas is inversely proportional to desertification. The areas of positive correlation (regression coefficient is 0~1) are mainly distributed in Kunming of central Yunnan near the Guizhou, central and eastern Chuxiong, Qujing City, central and southern Zhaotong City, and western Yunnan near southwest of Guiyang. For the more developed areas of the YGP, the environmental pollution brought by the development of the secondary and tertiary industries is not conducive to the restoration and sustainable land use, so the GDP is directly proportional to desertification in these areas. (3) The GWR regression coefficient between precipitation and desertification area is negatively correlated in most areas (**Fig 10**), but there are still some positive correlations for GWR regression coefficients in some areas. In the series results, the areas of negative correlation (regression coefficient -1~0) are mainly distributed in the Guiyang area, part of Bijie City, northwestern Yunnan, Zhaotong City, Zunyi City, Baoshan City, Linyi City, Pu'er City, and Chuxiongzhou. Combined with the precipitation data, it is found that the annual precipitation in these areas is only about 800mm. The increase of precipitation is conducive to vegetation growth and the growth of vegetation in turn increases the vegetation coverage, and the increase of vegetation coverage is conducive to the improvement of desertification. Therefore, in these areas, precipitation is negatively correlated with desertification. The areas of positive correlation (regression coefficient is 0~1) are mainly distributed in the Weinan and Weibei, Yuzhong, Qigui and western Yunnan areas. Combined with the precipitation data, the years of these areas are found. Where precipitation is abundant (higher than 1000mm a year), the water absorption capacity of vegetation and soil is saturated. The precipitation on the YGP is mostly instantaneous precipitation, and the intensity of short-term precipitation is great. Therefore, the increase of precipitation enhances the precipitation of these areas. The ability to wash has exacerbated desertification and soil erosion in the region. By Analyzing the GWR regression coefficient of the 10th period, during the period of 1989–2010 and 2016, the GWR regression coefficient of precipitation and desertification did not much change. However, in 2013, the absolute value of the GWR regression coefficient between precipitation and desertification increased significantly, and the main changes were concentrated in these areas, such as Kunshan, Zhaotong, Qujing, Qigui, Wenshan interior, southwestern Yunnan and southern Guizhou, and Baoxi, Nujiangzhou and Pu'er City in the west of the area, and the absolute values of the negative correlation coefficients increased significantly. Along with meteorological data, the **Fig 7** shows that during the period of 2010–2013, there was extreme drought with extreme precipitation scarcity in central Guizhou and the Guizhou-Guizhou-Chongqing areas. The decrease of precipitation destroyed the growth of vegetation and the vegetation coverage decreased significantly, leading to desertification in these areas. The situation is obviously worse. (4) The GWR regression coefficient between vegetation areas and WED desertification areas is negatively correlated in the most areas (**Fig 11**), but there are some positive correlations between GWR regression coefficients in partial areas. Among 10 series results, the areas with negative correlations (regression coefficient -1~0) are mainly distributed in Dehongzhou, Baoshan, Linyi, Pu'er, Yuzhong and Kunming near southwestern Guizhou Province. In the northern Qujing, Zhaotong, Wenshan, and Anshun, southwestern Guizhou, and Liupanshui. The areas of positive correlation are mainly concentrated in Diqingzhou and Nujiangzhou. There are also some sporadic distributions in other areas. The vegetation in the above areas is mainly plateau meadows. Meanwhile, due to other factors, such as the impact of precipitation on the desertification area, the regression coefficient between vegetation and desertification area shows a certain weak positive correlation. Accoring to the GWR regression coefficients of 10 periods between vegetation area and desertification area, the GWR regression coefficient between vegetation area and desertification area did not much change between 1989 and 2010, but the absolute value of the regression coefficients decreased significantly in 2013 in the following areas, such as Baoshan, Linyi, and Kunming and Qujing in the west of Yunnan. The GWR regression coefficient between vegetation and desertification area was negatively correlated, which is due to severe drought years between 2011 and 2013. So the vegetation insufficiently growth and obviously shrunk, and the areas of desertification is obviously expanded. In conclusion, the absolute value of the GWR regression coefficient of vegetation area and desertification area decreased significantly.

## Conclusion

In this study, long-term monitoring of WED was achieved on a large scale using time-series Landsat images, and the WED monitoring results show that the proposed method outperformed the other approaches in terms of OA and kappa. However, the classification accuracies of models based on Landsat images with medium spatial resolution remain limited because of the existence of mixed pixels and object spectral heterogeneity in large regions. In addition, we realized WED mapping of the YGP over the past 28 years from 1989 to 2016 based on the proposed method and analyzed the temporal and spatial variations of WED. Finally, the driving factors of WED on the YGP were analyzed based on a GWR model. The conclusions obtained are as follows.

The WED desertification on the YGP are mainly distributed in the Dianguiqian area, the Wumeng mountain area, and the border area of the western Yunnan and the area along the Red River. The driving factors of WED were analyzed based on the GWR model. We found that precipitation, vegetation area, and GDP have key roles in the processes of desertification reversion and development. These driving factors had different effects in different regions. The WED desertification in different areas has different causes due to environmental complexity of the region. The WED desertification in the Red River and Wumengshan areas is caused by carbonate lithology and geological structures, the Dianguiqian area is caused by geological factors such as poor basic lithology and climatic reasons. On the whole, The WED desertification areas on the YGP is increased from 1989 to 2016, but there are different in the different regions due to geographic environment complexity and some rocky desertification management projects from government. The proportion of the WED desertification coverage in most parts of Yunnan Province increased significantly in 2010–2013, especially in the central Yunnan region. The specific reason is that there is severe drought in Yunnan Province in 2012, so the precipitation is decreased, and the vegetation is destructed, finally, which leads to the WED desertification increasing. On the whole, the proportion of desertification in Guizhou Province is raised from 1989–2016, but the WED desertification areas do not changed significantly if there are abundant rainfall at same time.

These results provide meaningful information that the government can employ to prevent and control WED in the YGP. In future research, the integration of multi-source remote sensing data for long time-series desertification monitoring will be addressed and more indictors for building the desertification classification rule set will be considered.
